# Methicillin-Resistant *Staphylococcus aureus* ST80 Induce Lower Cytokine Production by Monocytes as Compared to Other Sequence Types

**DOI:** 10.3389/fmicb.2018.03310

**Published:** 2019-01-09

**Authors:** Fevronia Kolonitsiou, Matthaios Papadimitriou-Olivgeris, Anastasia Spiliopoulou, Eleanna Drougka, Eleni Jelastopulu, Evangelos D. Anastassiou, Iris Spiliopoulou

**Affiliations:** ^1^Department of Microbiology, School of Medicine, University of Patras, Patras, Greece; ^2^Division of Infectious Diseases, School of Medicine, University of Patras, Patras, Greece; ^3^Department of Public Health, School of Medicine, University of Patras, Patras, Greece

**Keywords:** *S. aureus*, MLST, PVL, toxic shock syndrome toxin, accessory gene regulator (*agr*), methicillin-resistant *S. aureus* (MRSA)

## Abstract

Methicillin-resistant *Staphylococcus aureus* (MRSA) remains an important cause of nosocomial and community-associated infections due to its ability to produce toxins and evade host’s immune responses. The aim of the present study was to investigate the association of monocytes immune response in terms of cytokines produced after inoculation with different MRSA clones. Thirty-one clinical MRSA strains were selected on the basis of clonal types, accessory gene regulator (agr) groups and toxin genes carriage. Isolates were identified as *S. aureus* by Gram stain, catalase, coagulase production and PCR for *nuc* gene. The presence of *mecA*, *lukS/lukF-PV* (Panton-Valentine Leukocidin) and *tst* (Toxic Shock Syndrome Toxin-1) genes, as well as, the determination of *agr* groups was performed by PCR. Clonality was investigated by means of multi-locus sequence typing (MLST). Peripheral blood mononuclear cells were stimulated with live bacterial cells for 45 min at a ratio of 1:10. Cells were incubated for 10 h and supernatants were collected. The levels of Tumor Necrosis Factor alpha (TNFa), IL-1b, IL-8, IL-6, IL-12p40, IL-10, interferon-gamma (IFN-γ) and IL-2, were measured by Human Cytokine Multiplex Immunoassay kit. Thirteen strains were *tst* and 12 *lukS/lukF-PV*-positive. Seven strains belonged to ST80 and ST225, five to ST30 and ST239, while the remaining seven isolates were grouped together as “other.” Strains belonging to ST80 induced statistically lower levels of TNFa, IL-1b, IL-8, IL-6, IL-10, IFN-γ, and IL-2. PVL-positive strains classified into ST80 clone induced statistically lower concentrations of most cytokines as compared to PVL-positive strains belonging to other clones, *tst*-positive strains and toxin-negative ones. Strains of *agr*3 group belonging to ST80 induced statistically lower concentrations of most tested cytokines as compared to *agr*3 strains not-belonging to ST80, *agr*2 or *agr*1. This low induction of immune response by MRSA ST80 cannot be attributed to the presence of neither *lukS/lukF-PV* nor *agr3*.

## Introduction

*Staphylococcus aureus* remains an important cause of infections, especially in skin and soft tissue, but can also provoke severe ones such as necrotizing pneumonia, bacteraemia and endocarditis (van Belkum et al., 2009). In Greece, *S. aureus* accounts for 9% of bacteraemias, while 40% of them are caused by MRSA (Kolonitsiou et al., 2017). MRSA was initially isolated from healthcare-associated infections causing outbreaks and gradually becoming endemic in the hospital setting with ST225 and ST239 being the most prominent clones (Drougka et al., 2014; Monecke et al., 2018). Since late 90s CA-MRSA arised worldwide and rapidly disseminated in the community (Witte, 2009; Drougka et al., 2014). The virulence of CA-MRSA is mainly due to the production of PVL which has a cytolytic activity on immune system’s cells (Witte, 2009; Drougka et al., 2014). In the Mediterranean area and especially in Greece such infections are mainly due to ST80 clone which during the last decade invaded the healthcare setting causing a large proportion of healthcare-associated infections (Witte, 2009; Drougka et al., 2014).

Asymptomatic nasal carriage of *S. aureus* is a common phenomenon, with approximately 50% of the general population being transient carriers and an additional 25% being persistent ones (von Eiff et al., 2001). Colonization is the prerequisite for invasive infection since most of infections are of endogenous origin caused by colonizing isolates (von Eiff et al., 2001). The capacity of several clones to disseminate can be partially due to their ability to evade host’s immune responses by secreting different immune evasion protein complexes, leading to persistent colonization increasing the risk of transmission (McCarthy and Lindsay, 2013). The main regulatory system of *S. aureus* that ensures adaptation of staphylococci to the environment acting as a quorum-sensing is the Agr system (Le and Otto, 2015). Up-regulation of virulence factors by Agr contributes to acute infection in several animal models, whereas, down-regulation by this system of microbial surface components has been implicated in enhanced biofilm formation and bacterial colonization. Among the Agr-controlled toxins are the pore-forming toxins, including the PVL (Le and Otto, 2015).

The aim of the present study was to investigate the association of monocytes’ immune response in terms of pro-inflammatory cytokines produced after inoculation with variable MRSA clones, classified into different *agr* groups.

## Materials and Methods

### Bacterial Isolates

Thirty-one clinical MRSA strains recovered from inpatients and outpatients with skin and soft tissue infections (SSTIs) or bloodstream infections (BSIs) at the University General Hospital of Patras during 2011–2014 were selected to be studied, representing the main clones identified in Greece, classified into different *agr* groups and carrying the genes of the superantigen Toxic Shock Syndrome Toxin-1 (TSST-1; *tst*) and the PVL (*lukS/lukF-PV*) (Table 1). The study was carried out under the Hospital Surveillance Program for multi-drug resistant bacterial colonization and infections and in accordance with the recommendations of the Ethics Committee of the University General Hospital of Patras that waived the need of inform consent from the patients with *S. aureus* infection that the strains were included in the study, whereas, a written informed consent was obtained, in accordance with the Declaration of Helsinki, from healthy volunteers from which PBMCs were collected. The study was approved by the Ethics Committee of the University General Hospital of Patras.

**Table 1:** Clinical and molecular characteristics of methicillin-resistant *S. aureus* strains included in the study.

ST	SCCmec	*agr*-allele	Toxin-genes	Type of infection
80 (7)	IV (7)	3 (5)	*tst*-negative, *lukS/lukF-PV*-positive (5)	SSTI (5)
		1 (1)	*tst*-negative, *lukS/lukF-PV*-positive (1)	SSTI (1)
		2 (1)	*tst*-negative, *lukS/lukF-PV*-positive (1)	SSTI (1)
225 (7)	II (7)	2 (4)	*tst*-positive, *lukS/lukF-PV*-negative (3)	SSTI (2), BSI (1)
			*tst*- and *lukS/lukF-PV*-negative (1)	SSTI (1)
		2 (2)	*tst*-negative, *lukS/lukF-PV*-positive (2)	SSTI (2)
		2 (1)	*tst*-positive, *lukS/lukF-PV*-negative (1)	BSI (1)
30 (5)	IV (5)	3 (5)	*tst*-positive, *lukS/lukF-PV*-negative (4)	SSTI (4)
			*tst*- and *lukS/lukF-PV*-negative (1)	SSTI (1)
239 (5)	III (5)	1 (5)	*tst*-positive, *lukS/lukF-PV*-negative (3)	SSTI (2), BSI (1)
			*tst*- and *lukS/lukF-PV*-negative (2)	SSTI (2)
5 (2)	IV (2)	1 (1)	*tst*- and *lukS/lukF-PV*-negative (1)	SSTI (1)
		2 (1)	*tst*- and *lukS/lukF-PV*-negative (1)	SSTI (1)
377 (2)	V (2)	1 (2)	*tst*-negative, *lukS/lukF-PV*-positive (2)	BSI (2)
22 (1)	IV (1)	2 (1)	*tst*-positive, *lukS/lukF-PV*-negative (1)	SSTI (1)
217 (1)	IV (1)	1 (1)	*tst*-positive, *lukS/lukF-PV*-negative (1)	SSTI (1)
770 (1)	II (1)	1 (1)	*tst*-negative, *lukS/lukF-PV*-positive (1)	SSTI (1)
**Control strains**			
Fri913		1	*tst*-positive, *lukS/lukF-PV*-negative	
Fri137		2	*tst*- and *lukS/lukF-PV*-negative	
ATCC49775		3	*tst*-negative, *lukS/lukF-PV*-positive	
HT20000195		4	*tst*- and *lukS/lukF-PV*-negative	


*Numbers of isolates are given in parentheses. ST, Sequencing Type; SCCmec, Staphylococcal Cassette Chromosome mec; SSTI, Skin and Soft Tissue Infection; BSI, Bloodstream Infection.*

### *S. aureus* Identification

Isolates were identified as *S. aureus* by Gram stain, catalase and coagulase production (Slidex Staph plus test, bioMerieux S.A., Marcy l’Etoile, France) and verified by molecular methods (PCR for *nuc* gene) (Zhang et al., 2004).

### Molecular Characterization

The presence of *mecA*, *lukS/lukF-PV* (PVL) and *tst* (TSST-1) genes as well as the determination of *agr* groups was performed by PCR (Jarraud et al., 2002). Positive control strains were: Fri 913 (*agr*1, *tst*-positive), Fri 137 (*agr*2), ATCC 49775 (*agr*3, *lukS/lukF-*PV*-*positive) and HT 20000195 (*agr*4) (Jarraud et al., 2002). The genes encoding the adhesins Fib (adhesin binding to fibrinogen), ClfA (adhesin binding to fibrinogen, involved in platelet aggregation and immune invasion), ClfB (adhesin binding to fibrinogen, cytokeratin 10, loricrin and involved in adhesion to epithelial cells and platelet aggregation), FnBPA (adhesin binding to fibronectin), Eno (encoding laminin binding protein) and EbpS (adhesin binding to elastin) were investigated by PCRs as described previously (Jarraud et al., 2002; Peacock et al., 2002; Tristan et al., 2003). Reference strains NCTC13552, Fri 913, ATCC119095, ATCC49775, ATCC31890, were used as controls. Clonality was investigated in all strains by MLST^1^. MRSA clones were defined according to their STs.

### Isolation of Human Peripheral Blood Mononuclear Cells

Peripheral blood mononuclear cells were isolated from buffy coats derived from blood donation bottles of six healthy volunteers by density centrifugation on Ficoll density gradient (Biochrom AG, Berlin, Germany) as previously described (Spiliopoulou et al., 2012). Briefly, collected mononuclear cells were washed in PBS and resuspended in RPMI-1640 medium supplemented with 10% heat-inactivated fetal calf serum (Biochrom AG) and 2 mM L-glutamine (HyClone), (CM). Cells at a density of 1 × 10^6^ cells/mL per well, were then seeded in 24-well flat bottom tissue culture plates (Sarstedt, Nümbrecht, Germany) and cultured at 37°C in a humidified, 5% CO_2_ atmosphere.

### Cytokine Measurement

PBMC (10^6^ cells/mL) were stimulated with live bacterial cells for 45 min at a ratio of 1:10, as in preliminary experiments, this ratio was proved to be the most efficient in cytokine production. (Spiliopoulou et al., 2012) Afterward, extracellular bacteria were lysed by lysostaphin (Sigma-Aldrich, St. Louis, MO, United States), and medium was replaced by CM supplemented with antibiotics. (Spiliopoulou et al., 2012) Cells were incubated for 10 h, since in preliminary experiment with the four control strains (Fri 913, Fri 137, ATCC 49775 and HT 20000195) in 2, 4, 6, 12, 24, and 48 h, all cytokines reached their peak between 6 and 12 h. Each experiment was carried out with mononuclear cells isolated from a single donor and performed in triplicate, with PBMCs from three donors; calculation of mean values was performed. Results for all 31 strains are based on at least three experiments from three different donors. LPS (10 ng/mL) was used as a positive control, and PBMCs without bacteria or LPS were used to assess spontaneous levels of cytokine secretion (negative control). Supernatants were collected, and the levels of TNFa, IL-1b, IL-8, IL-6, IL-12p40, IL-10, IFN-γ, and IL-2, were measured by Human Cytokine Multiplex Immunoassay kit (LINCO Research Inc., St. Charles, MO, United States) using Luminex^®^xMAPTM technology. A five-parameter regression formula was used to calculate the cytokine concentrations in samples from standard curves.

### Statistical Analysis

SPSS statistics version 23.0 (SPSS, Chicago, IL, United States) was used. Difference of cytokine production was assessed by two-tailed *t*-test. *p* < 0.05 was considered as statistically significant.

## Results

### Molecular and Clinical Characteristics of Isolates

All 31 strains were *mecA*-positive (MRSA). Twenty-six were from patients with SSTIs, whereas the remaining five were isolated from bactereamic patients. Toxin genes presence was verified in 25 strains; 13 were *tst*-positive and 12 *lukS/lukF-PV*-positive. No strain carried simultaneous both toxin genes. The adhesin genes were detected in most strains; *clfA* in 21 strains (68%), *clfB* and *ebpS* in 28 strains each (90%), *fib* and *eno* in 30 strains each (97%) and *fnbA* in 29 strains (94%). The main clones were ST80 (seven strains), ST225 (seven), ST30 (five), ST239 (five), while the remaining seven isolates, grouped together as “other STs,” belonged to ST5 (two), ST377 (two), ST22 (one), ST217 (one) and ST770 (one) (Table 1). Strains were classified into *agr*1 (11), *agr*2 and *agr*3 (10 strains each); no MRSA belonged to *agr*4 group. The presence of adhesin genes were not clonal related.

### Cytokine Measurement

**FIGURE 1:**
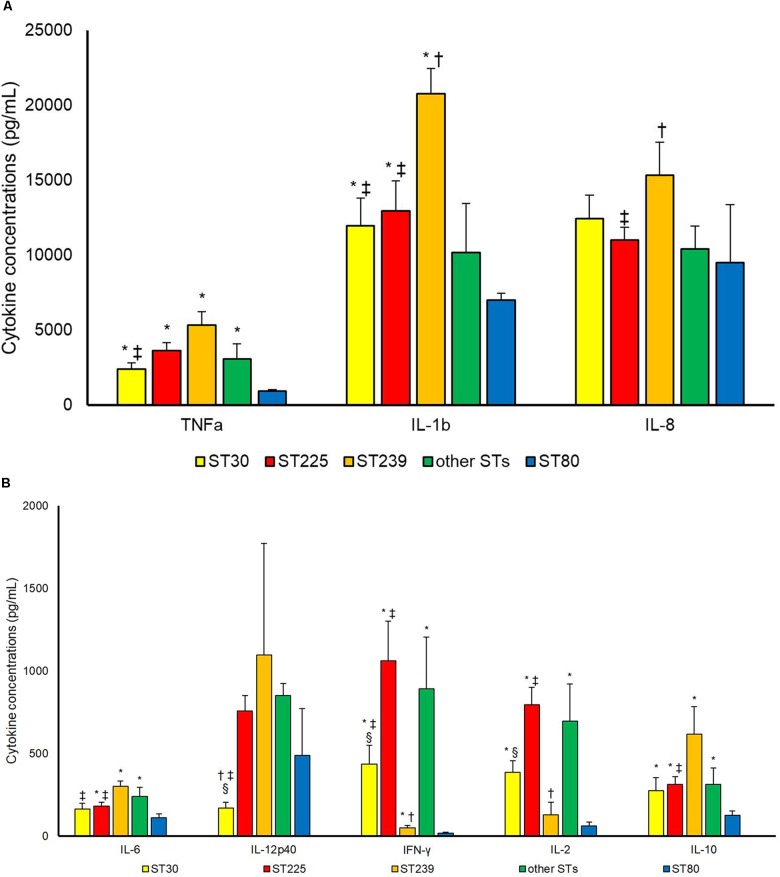
Cytokineconcentrations (pg/mL) **(A)** TNF-a, IL-1b, IL-8; **(B)** IL6, IL12p40, IFN-γ, IL-2, IL-10 in supernatant upon incubation of PBMCs with *S. aureus* cells. Differences between different Sequencing Types (STs) were assessed. *^∗^P* < 0.05 in comparison with ST80; ^†^*P* < 0.05 in comparison with other STs; ^‡^*P* < 0.05 in comparison with ST239; ^§^*P* < 0.05 in comparison with ST225.

Strains belonging to ST80 were compared to other STs concerning cytokine production in supernatants of PBMCs (Figure 1). ST80 induced statistically lower levels of TNFa [as compared to ST30 (*p* 0.002), ST225 (*p* < 0.001), ST239 (*p* < 0.001) and other STs (*p* 0.014)], IL-1b [as compared to ST30 (*p* 0.007), ST225 (*p* 0.002) and ST239 (*p* < 0.001)], IL-6 [as compared to ST225 (*p* 0.019), ST239 (*p* < 0.001) and other STs (*p* 0.014)], IFN-γ [as compared to ST30 (*p* 0.001), ST225 (*p* < 0.001), ST239 (*p* 0.040) and other STs (*p* 0.003)], IL-2 [as compared to ST30 (*p* < 0.001), ST225 (*p* < 0.001) and other STs (*p* 0.003)] and IL-10 [as compared to ST30 (*p* 0.042), ST225 (*p* 0.001), ST239 (*p* 0.003) and other STs (*p* 0.032)].

**FIGURE 2:**
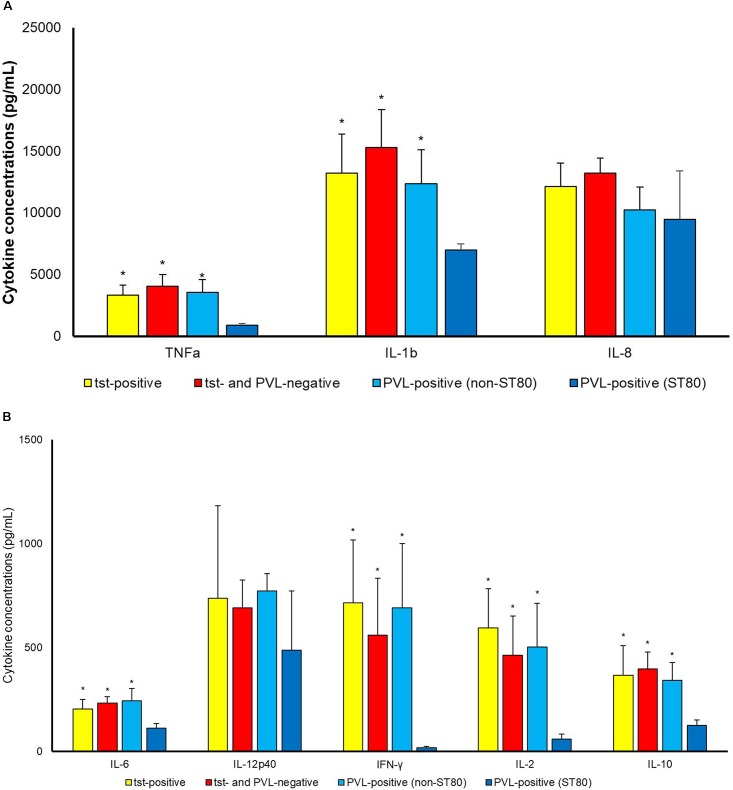
Cytokine concentrations (pg/mL) **(A)** TNF-a, IL-1b, IL-8; **(B)** IL6, IL12p40, IFN-γ, IL-2, IL-10 in supernatant upon incubation of PBMCs with *S. aureus* cells. Differences between *tst*-positive, *tst*- and PVL-negative and PVL-positive (belonging to ST80 and to STs other than ST 80) *S. aureus* were assessed. *^∗^P* < 0.05 in comparison with PVL-positive belonging to ST80.

As toxins were concerned (Figure 2), PVL-positive strains classified into ST80 induced statistically lower concentrations of TNFa (*p* 0.007), IL-1b (*p* 0.030), IL-6 (*p* 0.024), IFN-γ (*p* 0.015), IL-2 (*p* 0.019) and IL-10 (*p* 0.010) production as compared to PVL-positive strains belonging to other clones. ST80 PVL-positive clone induced also lower cytokine production as concerned to *tst*-positive strains [TNFa (*p* 0.001), IL-1b (*p* 0.019), IL-6 (*p* 0.027), IFN-γ (*p* 0.007), IL-2 (*p* 0.002) and IL-10 (*p* 0.043)] and toxin-negative ones [TNFa (*p* 0.001), IL-1b (*p* 0.004), IL-6 (*p* 0.002), IFN-γ (*p* 0.023), IL-2 (*p* 0.017) and IL-10 (*p* 0.002)]. No difference (*p* > 0.050) among *tst*-positive, non-ST80 PVL-positive and toxin-negative was observed.

**FIGURE 3:**
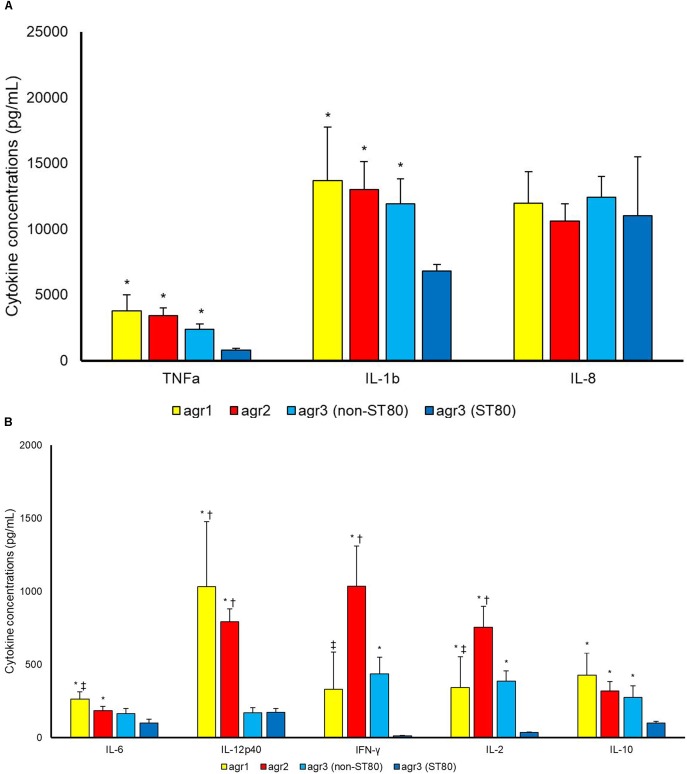
Cytokine concentrations (pg/mL) **(A)** TNF-a, IL-1b, IL-8; **(B)** IL6, IL12p40, IFN-γ, IL-2, IL-10 in supernatant upon incubation of PBMCs with *S. aureus* cells. Differences between *agr*1-positive, *agr*2-positive and *agr*3-positive (belonging to ST80 and to STs other than ST80) *S. aureus* were assessed. *^∗^P* < 0.05 in comparison with *agr*3-positive belonging to ST80. ^†^*P* < 0.05 in comparison with *agr*3-positive belonging to ST other than ST80. ^‡^*P* < 0.05 in comparison with *agr*2-positive.

Strains of *agr*3 group belonging to ST80 induced statistically lower concentrations of most tested cytokines as compared to *agr*3 strains not-belonging to ST80 [TNFa (*p* 0.005), IL-1b (*p* 0.018), IFN-γ (*p* 0.003), IL-2 (*p* 0.001) and IL-10 (*p* 0.042)], *agr*2 [TNFa (*p* < 0.001), IL-1b (*p* 0.008), IL-6 (*p* 0.019), IL-12p40 (*p* < 0.001), IFN-γ (*p* 0.001), IL-2 (*p* < 0.001) and IL-10 (*p* 0.003)] or *agr*1 types [TNFa (*p* 0.017), IL-1b (*p* 0.042), IL-6 (*p* 0.003), IL-12p40 (*p* < 0.001), IL-2 (*p* 0.002) and IL-10 (*p* 0.035)] (Figure 3).

## Discussion

ST80 has been proven an extremely successful MRSA clone in the Mediterranean area (Drougka et al., 2014). In Greece it represents the predominant clone among CA-MRSA, accounting for more than 85% of the isolates, while since 2008 a gradual increase of healthcare-associated MRSA infections was caused by this clone, highlighting its capacity of dissemination in the community and the hospital setting (Witte, 2009; Drougka et al., 2014). This dissemination and capacity to colonize could indicate a propensity in evading host defenses.

CA-MRSA ability to colonize and infect healthy individuals and provoke severe infection is partially due to production of PVL. PVL triggers the production of IL-8 by neutrophils and of IL-1b by monocytes and macrophages (Perret et al., 2012). PVL and TSST-1 remain important for the recruitment of neutrophils having a clear proinflammatory cytokine response. (Yoong and Pier, 2012). In our study, there are conflicting results concerning PVL-positive strains. PVL-positive strains not belonging to ST80 clone had similar cytokine response as compared to PVL*-*negative strains, while PVL-positive strains belonging to ST80 showed a significantly lower cytokine production by PBMC as compared to the aforementioned groups.

TSST-1 is another important toxin belonging to the category of staphylococcal superantigens and is primarily responsible for toxic shock syndrome development, by triggering a massive release of proinflammatory cytokines producing an overwhelming inflammatory response (Nakagawa et al., 2005; Xu and McCormick, 2012). In a rabbit model of infective endocarditis and sepsis, comparing different lineages of *S. aureus*, even though no difference in lethality was observed among investigated clones, strains carrying *tst* gene caused lethal sepsis (King et al., 2016). In the present study, pro-inflammatory immune response of PBMC *tst*-positive MRSA isolates did not differ from those that did not carry *tst* gene.

Panton-Valentine Leukocidin, TSST-1 and other virulence factors’ production is regulated mainly by the *agr* system, which is also responsible for adaption of individual toxin production according to the phase of colonization and infection (Cheung et al., 2011; Le and Otto, 2015). We found that isolates carrying *agr*3, especially those belonging to ST80, induced lower levels of cytokine concentrations in monocyte supernatant as compared to those carrying *agr*1 and *agr*2. In murine models of *S. aureus* pneumonia and bacteraemia, deletion of *agr* resulted in better survival (Heyer et al., 2002; Bubeck Wardenburg et al., 2007), while the presence of *agr*2 resulted in higher mortality in MRSA bacteraemia (Cechinel et al., 2016). Our hypothesis is that ST80 *agr*3 induces lower virulence factor production, including PVL, resulting in lower pro-inflammatory cytokine induction, but more studies are needed to elucidate this correlation.

The onset and duration of *S. aureus* colonization is dependent of immune system response. A combined induction of multiple pro-inflammatory chemokines and cytokines, including IL-1β, IL-6, TNF-α, IFN-γ, IL-8, IL-12, is necessary to clear *S. aureus* from human nasal mucosa (Cole et al., 2016). The host’s failure to amount an elaborative combined response can lead to persistent colonization (Cole et al., 2016). As shown in the present study, strains belonging to ST80 induced significantly lower production of most of aforementioned pro-inflammatory mediators as compared to other STs (ST30, ST239, ST225), favoring colonization.

As it was previously shown, IFN-γ plays an important role in controlling staphylococcal colonization (Satorres et al., 2009). It enhances pro-inflammatory response of human mast cells in response to *S. aureus* by generating reactive oxygen species which leads to bacterial killing (Swindle et al., 2015). As macrophages are concerned, IFN-γ increases their phagocytic response and accelerates *S. aureus* killing (Greenlee-Wacker and Nauseef, 2017). Another role of IFN-γ is that confers resistance of keratinocytes to *S. aureus* alpha toxin induced cell death (Brauweiler et al., 2016). In the present study, ST80 induces a significantly lower IFN-γ production by monocytes as compared to ST30, ST239, and ST225, which can partially explain its ability to evade host immune response and promote chronic colonization (Prabhakara et al., 2011).

The present study has several limitations. First, we did not measure the level of *tst* and *lukS/lukF-PV* genes expression or *agr* activity. Secondly, mRNA expression levels of cytokines were not performed. Even though the strains included in the present study belonged to globally disseminated clones, other clones such as ST8 or ST1, which are the predominant in North America were not included (Witte, 2009). Moreover, even though the adhesin genes genetic background of the strains was determined, no adhesion activity of each MRSA clone to PBMCs was evaluated. The study is an *in vitro* one, so studies in murine models and in colonized or infected patients are needed to elucidate the immune response *in vivo* of different clones and especially ST80. We did not include isolates carrying the *agr*4 system since these isolates were rarely detected in our setting.

## Conclusion

In conclusion, MRSA strains belonging to ST80 clone induce lower level of pro-inflammatory cytokines production by monocytes as compared to all other clones. This difference cannot be attributed neither to the presence of *lukS/lukF-PV* genes nor to *agr*3 system that are usually present in ST80. This low induction of immune response by MRSA ST80 probably leads to evasion of hosts’ immune defenses and can partially explain its silent and successful dissemination in the community and the hospital setting in Greece.

## Author Contributions

IS, EA, and FK designed the experiments. FK, AS, and ED performed the experiments. FK, MP-O, and EJ collected the data. MP-O, FK, EJ, and IS performed the analysis. MP-O and FK wrote the manuscript. All authors contributed to manuscript revision, read and approved the submitted version.

## Conflict of Interest Statement

The authors declare that the research was conducted in the absence of any commercial or financial relationships that could be construed as a potential conflict of interest.

## Footnotes

1 http://saureus.mlst.net/misc/info.asp

## ABBREVIATIONS

Agr: accessory gene regulator
BSI: bloodstream infection
CA-MRSA: community-acquired methicillin-resistant *S. aureus*
CM: complete medium
IFN-γ: interferon-gamma
IL: interleukin
LPS: Lipopolysaccharide
MLST: Multi Locus Sequence Typing
PBMC: Peripheral blood mononuclear cell
PVL: Panton-Valentine Leukocidin
SSTI: skin and soft tissue infection
ST: sequence type
TNFa: Tumor Necrosis Factor alpha
TSST-1: Toxic Shock Syndrome Toxin-1
